# Economic and sociodemographic inequalities in complete denture need among older Brazilian adults: a cross-sectional population-based study

**DOI:** 10.1186/s12903-016-0233-9

**Published:** 2016-07-04

**Authors:** Daniela Mendes da Veiga Pessoa, Angelo Giuseppe Roncalli, Kenio Costa de Lima

**Affiliations:** Department of Dentistry, University of State of Rio Grande do Norte, Caicó, Brazil

**Keywords:** Brazil, Health inequalities, Aged, Socioeconomic status, Complete dentures

## Abstract

**Background:**

Brazil has severe socioeconomic inequalities, resulting in major oral health problems for the Brazilian elderly, such as tooth loss and, consequently, a need for oral rehabilitation. The aim of this study was to evaluate inequalities in complete denture need among older Brazilian adults in relation to social determinants at individual and contextual levels.

**Methods:**

This retrospective study was based on data from the national oral health survey, SB Brasil 2010, in which 7,619 older adults aged 65–74 years participated. The dependent variable was complete denture need. The independent variables at the first level were age, sex, race, and socioeconomic status. The independent variables that were used to identify inequality at the contextual level were geographic region of Brazil, the population of the municipality where the subject lived, whether the subject lived in the state capital or not, and the Human Development Index. In order to describe the socioeconomic characteristics, a socioeconomic cluster variable was created using the multivariable cluster analysis technique. Prevalence ratios (PRs) with 95 % confidence intervals (CIs) were determined to evaluate the effect of each variable. Two-level multivariable modeling was performed to examine the individual and contextual effects.

**Results:**

There was a high prevalence of complete denture need among older Brazilian adults. The main factors associated with the prevalence of complete denture need were individual socioeconomic status (PR: 1.81; 95 % CI: 1.65–1.99), and the city-level contextual effect (PR: 1.20; 95 % CI: 1.08–1.34).

**Conclusions:**

Consistently poor rates of oral health rehabilitation were found among older Brazilian adults, and were associated with significant social inequality. Complete denture need was strongly associated with individual socioeconomic position. It was also verified that the Human Development Index, the city-level contextual effect, was associated with complete denture need.

## Background

In recent years, global demographic and epidemiological transitions have led to a significant increase in the elderly population, especially in developing countries. As a result, there is a growing demand for public health policies and investment in healthcare for this population group. Oral health and dental care are important aspects of health. Oral health is currently experiencing a global epidemiological transition, with decreasing rates of edentulousness [1]. In Brazil, however, the situation regarding edentulousness appears relatively unchanged, with the most recent epidemiological surveys of oral health revealing a high prevalence of tooth loss. In the 1980s and 1990s, a combination of improvements in socioeconomic conditions, especially in education, fluoridation of tap water, and widespread use of fluoride toothpaste, contributed to a reduction in the prevalence of dental caries, the main cause of tooth loss, but the elderly assessed in this study (in 2010) did not benefit from these measures during their own childhood and adolescence [2].

The loss of teeth without replacement with dentures results in a severe loss of oral function. This condition is common among poor populations in both developed and developing countries [3]. In addition, it has been suggested that the association between tooth loss without replacement prostheses and malnutrition in the elderly is related to selective food intake because of limited chewing ability [4, 5]. According to the World Health Organization (WHO), health inequalities are avoidable inequalities in health between groups of people within countries and between countries, and these inequalities arise from inequalities within and between societies. Social and economic conditions and their effects on people’s lives determine their risk of illness [6]. In accordance with this point of view, Marmot [7] highlighted that “health inequalities are perhaps the most damning indictments of social and economic inequalities”.

Brazil is a country marked by severe socioeconomic inequalities, leading to disparities in dental health. Tooth loss and, consequently, oral rehabilitation need are important oral health concerns in the Brazilian elderly [8]. The individual determinants of oral diseases have been well documented, but it is important to determine the social and geographic contextual influences on these oral health problems [8, 9]. There are 20.6 million older adults in Brazil, representing 10.8 % of the population according to the last census conducted in Brazil in 2010 [10]. Rates of tooth loss among this group are high, which may be the result of insufficient dental care and limited access to oral health services, indicating that there is huge unmet need for treatment with dentures and restorative dental care among older adults.

Therefore, an understanding of denture need among older adults in Brazil at individual and contextual levels will allow a greater understanding of the extent to which geographical and economic differences can influence the demand for dentures, and help in the formulation of a more equitable oral health policy. According to sociocultural epidemiology theory, a new multidimensional analysis of health is needed to examine the links between health and disease using a multilevel approach [11, 12].

The aim of the present study was to evaluate inequalities in complete denture need among older Brazilian adults in relation to social determinants at the individual and contextual levels. In addition, the results of the present study may aid in the planning, monitoring, and evaluation of oral health services in Brazil.

## Methods

### Study population

This retrospective study was based on data from the national epidemiological survey of oral health, Project SB Brasil (*Projeto Saúde Bucal Brasil* – Project Oral Health Brazil), which was carried out in 2010. It was a cross-sectional study that included elderly Brazilian subjects aged 65–74 years. The design of SB Brasil, a multistage cluster study, has been previously described [13, 14]. The sample domains were the state capitals and non-capital municipalities in the five regions of Brazil. The sample units were census zones and households in the state capitals, and census zones and households in municipalities of the non-capitals. A total of 30 sectors in each capital and 30 non-capital municipalities of each region were randomly selected. The sample considered fields clustered according to density of the total population and the internal variability of the indices. The final sample was representative of each state capital and federal district in Brazil. From these criteria, a total of 7,619 older adults from municipalities in the state capital and non-capital cities from all five geographic regions of Brazil participated in the study.

The SB Brasil 2010 Project was approved by the Research Ethics Committee of the Ministry of Health and was registered with the National Commission of Research Ethics under number 15.498.

### Sources of data

Data on complete denture need as well as and socioeconomic and sociodemographic variables were obtained from Project SB Brasil 2010 and databases of the National Demographic Census (www.ibge.gov.br and www.pnud.org.br), carried out in 2010 by the Brazilian Institute of Geography and Statistics.

### Dependent variable

The indices used in SB Brasil 2010 to assess the oral health status of older adults met the recommendations of the 4th Edition of the WHO Instruction Manual for Basic Epidemiological Surveys in Oral Health [13], and took into account experience accumulated in previous surveys in several regions of Brazil, particularly from 1980. The indices of SB Brasil included the following: upper denture need (none/one denture element/more than one prosthesis element/combination of dentures/complete denture) and lower denture need (none/one denture element/more than one prosthesis element/combination of dentures/complete denture). The category selected for this study was “complete denture”, and the dependent variable in this study was “complete denture need”, i.e., requiring complete dentures in one or both dental arches.

### Independent variables

The individual variables were age (65–69 years/70–74 years), sex (male/female), race (white/mixed), and socioeconomic status (high/low), obtained from a questionnaire administered by public health service dentists to participants in their homes. Race was assessed through self-report according to the classification proposed by the Brazilian Institute of Geography and Statistics.

A socioeconomic status variable was constructed using the cluster analysis technique, and included: 1) the number of consumer goods (e.g., televisions, refrigerators, stereos, microwaves, telephones, cell phones, washing machines, dishwashers, personal computers, and cars), ranging from 0 to 11; 2) family income (monthly family income in Brazilian currency, *reais*); 3) years of education; and 4) household crowding (number of people living in the residence of the older adult divided by the number of rooms serving as bedrooms in the residence) [13].

The city-level sociodemographic variables were: population size (municipalities up to 20,000 inhabitants, 20,001–100,000 inhabitants, 100,001–500,000 inhabitants, and more than 500,000 inhabitants); type of municipality (capital or non-capital); region of Brazil (north, northeast, center-west, southeast, south); and Human Development Index (HDI: up to 0.762; 0.763 and over). The HDI is a composite measure encompassing information on income, education, and life expectancy, and is used to assess the social development of an area and to compare quality of life on an international basis [15]. The HDI was taken from the “Human Development Atlas” provided by the Brazilian agency of the United for Nations Development Program (www.pnud.org.br). The same method was used for all municipalities in Brazil [8]. HDI scores range from 0 to 1, and the higher the value, the better the social development. In the present study, the HDI variable was dichotomized according to the median value.

The city-level variables were population size, type of municipality, and region of Brazil and were extracted from the National Demographic Census, carried out in 2010 by the Brazilian Institute of Geography and Statistics.

### Data analysis

A descriptive analysis of the dependent, individual, and contextual independent variables was performed. A Poisson robust regression model was used for bivariate analysis to obtain crude prevalence ratios (PRs) and 95 % confidence intervals (CIs). In multivariate cluster analysis, a hierarchical technique was first used to select the number of clusters and profile cluster centers to serve as initial cluster seeds (centroid elements) in the nonhierarchical procedure. Following this, hierarchical process analyses produced dendrograms that resulted in the choice of two socioeconomic clusters. The between groups linkage clustering algorithm was used in the agglomerative hierarchical method, with Euclidean simple used as a distance measure, as this was used in the nonhierarchical part of the analysis. Ward’s clustering algorithm was used to check the stability of clusters at a 2^nd^ time stage (after initial analysis).

The nonhierarchical technique was selected because of the large sample size. From the final cluster model, based on standardized values (by Z score), the presence of two clusters was observed: one composed of individuals with high socioeconomic status, and the other composed of individuals with low socioeconomic status.

A two-level multilevel mixed-effect Poisson regression analysis was performed to verify the effect of individual characteristics and also the context’s influence on the outcome. In this case, the context was represented by one level of aggregation, taking into account the Brazilian administrative organization.

Exploratory analysis was performed to evaluate the effect of the level through calculation of PRs with respective 95 % CIs, with the better situation as the reference category. In addition, two-level multivariable modeling was constructed for the outcome using significant explanatory variables. The analysis started with a random intercepts model (null model) in order to verify whether the contextual effects were significant. The final model was executed when the variables were added and the model remained significant. Only HDI was used as a contextual variable in the final model because it was collinear with the other contextual variables, and also included the respective municipality.

## Results

The majority of the study population studied were female, were aged 65–69 years, were mixed race, resided in Brazilian cities in the northeast of Brazil, resided in municipalities with low HDI, and had low economic status. In terms of oral health rehabilitation, it was observed that a high percentage of older adults needed complete dentures (Table 1).

**Table 1:** Description of the oral health and social demographics characteristics of older people in Brazil

	Number	Percent
Complete denture need		
No	4,204	56.0
Yes	3,299	44.0
Age		
65–69 years	4,318	56.7
70–74 years	3,301	43.3
Gender		
Male	2,903	38.1
Female	4,716	61.9
Race		
White	3,577	48.2
Mixed	3,849	51.8
Socioeconomic status		
Low	5,406	71.5
High	2,158	28.5
Region		
North	1,758	23.1
Northeast	2,294	30.1
Southeast	1,287	16.9
Centerwest	1,117	14.7
South	1,163	15.3
Population size		
Up to 20,000 inhabitants	491	6.4
20,001–100,000 inhabitants	647	8.5
100,001–500,000 inhabitants	1,846	24.2
More than 500,000 inhabitants	4,635	60.8
Type of municipality		
Capital	6,003	78.8
Non-capital	1,616	21.2
HDI		
0.763 and over	4,007	52.8
Up to 0.762	3,612	47.4

There was a higher prevalence of complete denture need among female older adults, those aged 70–74 years, and those of mixed race. There was greater complete denture need among those residing in the north and northeast regions of Brazil than among those in the south. The need was also higher in older adults living in non-capital municipalities, in municipalities with small populations, and among those with a poorer socioeconomic status and HDI (Table 2).

**Table 2:** Bivariate associations between complete denture need and the independent variables according the levels

	Complete denture need			*p*-value
	*n*		PR(CI 95 %)	
Individual level		Yes		
*Age*				
65–69 years	4,253	41.5(40.7;42.3)	Ref	
70–74 years	3,250	47.2(45.6;48.8)	1.14(1.08;1.19)	<0.001
*Gender*				
Male	2,848	40.8(39.3;42.2)	Ref	
Female	4,655	45.9(44.6;47.2)	1.12(1.06;1.18)	<0.001
*Race*				
White	3,518	38.8(37.5;40.0)	Ref	
Mixed	3,792	48.6(47.1;50.1)	1.25(1.19;1.32)	<0.001
*Socioeconomic status*				
High	2,115	26.3(25.2;27.4)	Ref	
Low	5,335	50.9(49.5;52.2)	1.93(1.79;2.08)	<0.001
City level				
*Region*				
North	1,721	53.7(51.2;56.2)	1.76(1.60;1.94)	<0.001
Northeast	2,266	42.9(41.2;44.6)	1.40(1.27;1.55)	<0.001
Southeast	1,277	39.8(37.6;41.9)	1.30(1.17;1.46)	<0.001
Centerwest	1,091	50.0(47.1;52.9)	1.64(1.47;1.82)	<0.001
South	1,148	30.5(28.8;32.2)	Ref	
*Population size*				
Up to 20,000 inhabitants	484	56.8(51.8;61.8)	1.42(1.30;1.52)	<0.001
20,001–100,000 inhabitants	644	54.2(50.1;58.3)	1.36(1.25;1.47)	<0.001
100,001–500,000 inhabitants	1,805	47.3(41.2;49.4)	1.18(1.12;1.26)	<0.001
More than 500,000 inhabitants	4,750	39.9(38.8;41.0)	Ref	
*Type of municipality*				
Capital	5,911	41.6(40.6;42.6)	Ref	
Non-capital	1,592	52.8(50.2;55.4)	1.27(1.20;1.34)	<0.001
*HDI*				
0.763 and over	3,954	38.0(36.8;39.2)	Ref	
Up to 0.762	3,549	50.6(48.9;52.2)	1.33(1.26;1.40)	<0.001

*CI* confidence interval, *PR* prevalence ratio

The outcome was fitted in a null model in order to examine the contextual effects. Multilevel Poisson regression and the likelihood ratio (LR) were used to identify which contextual variables were associated with complete denture need (Table 3).

**Table 3:** Fixed and random effects parameters in the multilevel mixed-effect Poisson regression analysis for the null model

Complete denture need		
Fixed effects	Intercept	95 % (CI)
City level	−0.76	−0.82;−0.69
Region level	−0.85	−1.02;−0.68
Population size level	−0.72	−0.86;−0.58
Type of municipality level	−0.76	−0.93;−0.59
Random effects	Variance (SE)	LR Test (Chi^2^;*p*)
City level	0.06(0.01)	84.1;<0.001
Region level	0.04(0.02)	78.5;<0.001
Population size level	0.02(0.01)	38.1;<0.001
Type of municipality level	0.01(0.01)	25.14;<0.001

*CI* confidence interval, *SE* standard error, *LR* likehood ratio

Based on these results, a multilevel analysis, including the HDI as the contextual level for complete denture need, was performed as shown in Table 4. It was observed that, in the final model, most of the contextual variables remained significant with a slight adjustment in the PR. The city-level variance dropped from 0.038 to 0.021 (44.7 %) indicating a large effect. The *p*-value for the LR test in model 2 indicated that it was the most appropriate variable to describe the individual and contextual effects.

**Table 4:** Multilevel mixed-effect Poisson regression analysis for the complete denture need

	Complete denture need			
	Model 1 (*n* = 7,450)		Model 2 (*n* = 7,450)	
	PR(95 % CI)	*p*-value	PR(95 % CI)	*p*-value
Individual level				
Gender				
Female	1.12 (1.06;1.18)	<0.001	1.14(1.06;1.22)	<0.001
Age				
70–74 years	1.12 (1.07;1.18)	<0.001	1.12(1.05;1.20)	0.001
Race				
Mixed	1.01 (0.99;1.03)	0.053	1.00(0.98;1.03)	0.519
Socioeconomic status				
Low	1.91 (1.77;2.07)	<0.001	1.81(1.65;1.99)	<0.001
City level				
HDI			1.20(1.08;1.34)	<0.001
Fixed effects				
Intercept (95 % CI)	−1.39 (−1.51;−1.28)		−1.51(−1.63;−1.39)	
Random effects	Variance (95 % CI)		Variance (95 % CI)	
City level	0.038 (0.02;0.07)		0.021(0.009;0.049)	
LR test (Chi^2^, *p*-value)	39.76;<0.001		18.10;<0.001	

*PR* prevalence ratio, *CI* confidence interval, *HDI* human development index. Model1 for the individual variables only, model 2 for individual + city

## Discussion

This study found a high prevalence of complete denture need among the elderly Brazilian population, and the main factors implicated were related to individual socioeconomic position and a city-level contextual effect.

The most recent epidemiological surveys on oral health in Brazil revealed that the poorest geographic regions of the country, municipalities in the countryside, and those with a smaller population had the poorest oral health indicators [13, 16]. In a study that aimed to evaluate social determinants of dental treatment needs in Brazilian adults, it was observed that prosthetic treatment need was associated with individual and contextual levels, and adults living in cities with better quality of life (higher HDI) had a lower requirement for dental treatment [8]. These findings are consistent with those of the present study.

In examining the basis for Brazilian socioeconomic inequalities, it is first necessary to understand that both poverty and levels of social inequality do not arise from insufficient resources, but from historical processes. Brazil has been undergoing major economic, social, and demographic transformation, especially over the last four decades, with an accelerated redistribution of the economically active population from the agricultural sector to industry and services, resulting in rapid urbanization, a decrease in fertility rate, and an improvement in the HDI (0.649 in 1975 and 0.800 in 2005). These trends significantly influenced the lives and working conditions of the population and, consequently, their health conditions. However, Brazil developed economically because of its very diverse natural environment, and the extraordinary growth in wealth and modernization of the economy did not translate into an improvement in wealth distribution [17].

In terms of the relationship between the socioeconomic profile and oral health status, evidence shows that socioeconomic factors may be associated with different approaches to use of dental services. The present study showed that a substantial proportion of the older adult population needed dentures, and may indicate that individuals have gaps in their dental treatment, such as a lack of restorative treatment, implants or fixed partial dentures [18]. In 2004, Brazil created the National Oral Health Policy, with the intention of improving access to dental care and the oral health status of the population. This strategy focused primarily on changing the dental care model, which was previously concerned with tooth extraction and treatment of schoolchildren, to a model aimed at reorienting concepts and practices in the field of oral healthcare to provide a new working process to provide better nationwide care. This policy included health promotion and protection strategies, diagnosis and treatment of dental diseases, and dental rehabilitation. For the latter, Specialized Dental Centers and Regional Laboratories of Dental Prostheses (RLDP) were established in order to widen and improve secondary dental care. These RLDP aimed to address oral rehabilitation, recognized as a major requirement for the population, and may lead to a reduction in demand for complete dentures [19, 20].

Brazilian Health System data shows that there was a significant increase in the production of dental prostheses between the 2006 and 2013, from 60,286 to 471,089 dentures [21]. However, considering that oral diseases among older adults in Brazil are characterized by high tooth loss and low rehabilitation, and that these losses occur throughout life, it is likely to take time for new generations of Brazilian children and adolescents to become dentate adults and older adults, when there should be an impact on the epidemiological data.

Fixed and removable dentures are commonly used to replace missing teeth in order to improve chewing, esthetics, and pronunciation. Despite these favorable effects, not all people who lose their teeth use dentures [22]. Reasons include the difficulty of access to dental care for the elderly as well as cultural factors. Moreira et al. identified low education levels, low income, and scarce availability of public oral health services as barriers to use of oral health care services among the Brazilian elderly. Martins et al. also observed that the aged appear to minimize the importance of oral health issues in comparison to their other health problems [23, 24].

Regarding inequalities in oral rehabilitation in Brazil, Aguiar and Celeste observed that the greatest need for complete dentures was in the north (7.2 %), which also showed the lowest use among all regions (9.71 per 100,000). In general, these authors found that the increase in the number of RLDP and production of prostheses differed among the regions, and may reflect differences in demand for prosthetic rehabilitation in Brazil [25].

A Brazilian epidemiological survey on oral health in 2003, which aimed to investigate the association between sociodemographic and contextual dental services and the need for full prosthesis among elderly Brazilians, revealed that the risk of needing a full prosthesis was lower among individuals living in the southern region of Brazil than in the northern region (odds ratio (OR): 0.67; 95 % CI: 0.48–0.94) and higher in municipalities with lower educational levels than in those with higher educational levels (OR: 1.57; 95 % CI: 1.09–2.27) [26]. Mariño and Giacaman (2014) assessed the oral health status and treatment needs of an ambulant elderly population living in the Maule Region, Chile, and provided descriptive information on their distribution according to selected sociodemographic characteristics. They found that a high proportion of those who needed dental prosthetic appliances had unmet prosthetic needs (72.0 %), and that the level of education was a significant predictor. Those with primary education were 1.6 times more likely to have unmet prosthetic needs than participants with higher levels of education [27]. According to Nadgere et al. [28], social pressure to maintain esthetics and function can be a driving force in influencing individuals in upper socioeconomic classes to have their missing teeth replaced. In addition, attitude to, and awareness of, the necessity for dental care, and the cost of dental treatment can also be important factors in determining the denture status of an individual.

In addition to strategies that could improve oral health in the elderly, such as expansion of specialized care, a focus on avoidable damage is required, in accordance with the Brazilian Oral Health Policy strategies, to prevent future damage to a future generation of older adults. These strategies include fluoridation of public water supplies in municipalities with higher socioeconomic deprivation, implementation of oral health teams in the Family Health Strategy (the Brazilian model for primary care), and collaborative actions between schools and primary care through the “Health at School” Program.

### Limitation

It should be noted that the cross-sectional design does not allow analysis of the cause-effect relationship between the variables studied.

## Conclusions

The present study found a low prevalence of oral health rehabilitation among older Brazilian adults, as shown by the great need for complete dentures, especially among those from low socioeconomic strata. Complete denture need, related to secondary care, was strongly associated with individual socioeconomic position in Brazilian older adults. It was also verified that HDI, the city-level contextual effect, was a factor associated with the need for complete dentures. To provide a more equitable and decisive oral health care model for older adults across all municipalities of Brazil, each of which has its own socioeconomic, geographical and cultural profile, different oral health care strategies are urgently required to promote oral health and the availability and use of dental services.

## Abbreviations

CI, Confidence interval; HDI, Human Development Index; LR, Likelihood ratio; OR, Odds ratio; PR, Prevalence ratio; RLDP, Regional Laboratories of Dental Prosthesis; WHO, World Health Organization
